# Time-dependent bending rigidity and helical twist of DNA by rearrangement of bound HU protein

**DOI:** 10.1093/nar/gkt593

**Published:** 2013-07-04

**Authors:** Binu Kundukad, Piewen Cong, Johan R. C. van der Maarel, Patrick S. Doyle

**Affiliations:** ^1^BioSystems and Micromechanics (BioSym) IRG, Singapore MIT Alliance for Research and Technology (SMART), Singapore 138602, ^2^Singapore-Massachusetts Institute of Technology Alliance, National University of Singapore, Singapore 117576, ^3^Department of Physics, National University of Singapore, Singapore 117542 and ^4^Department of Chemical Engineering, Massachusetts Institute of Technology, Cambridge, MA 02139, USA

## Abstract

HU is a protein that plays a role in various bacterial processes including compaction, transcription and replication of the genome. Here, we use atomic force microscopy to study the effect of HU on the stiffness and supercoiling of double-stranded DNA. First, we measured the persistence length, height profile, contour length and bending angle distribution of the DNA–HU complex after different incubation times of HU with linear DNA. We found that the persistence and contour length depend on the incubation time. At high concentrations of HU, DNA molecules first become stiff with a larger value of the persistence length. The persistence length then decreases over time and the molecules regain the flexibility of bare DNA after ∼2 h. Concurrently, the contour length shows a slight increase. Second, we measured the change in topology of closed circular relaxed DNA following binding of HU. Here, we observed that HU induces supercoiling over a similar time span as the measured change in persistence length. Our observations can be rationalized in terms of the formation of a nucleoprotein filament followed by a structural rearrangement of the bound HU on DNA. The rearrangement results in a change in topology, an increase in bending flexibility and an increase in contour length through a decrease in helical pitch of the duplex.

## INTRODUCTION

The chromosome of most bacteria consists of a single closed circular DNA molecule of considerable size. For instance, the circular genome of *Escherichia coli* contains 4950 ± 250 kb and has a contour length of 1.6 mm. This single DNA molecule is accommodated inside a cell of ∼1 μm diameter and 3–5 μm length. Bacterial chromosomal DNA is hence about a thousand times compacted and occupies ∼25% of the cellular volume (1,2). This compaction is facilitated by negative supercoiling with the help of enzymes such as topoisomerase and gyrase, but nucleoid associated proteins (NAPs), macromolecular crowding and confinement by the cell envelope are also thought to play a role (3–6).

The functioning of NAPs such as HU, IHF, HNS and Fis is not clearly understood, but it has been shown that they induce structural changes on DNA (7–11). HU is one of the most abundant NAPs found in bacteria and plays a role in many cellular processes like the compaction, transcription and replication of the genome (12–14). It is present throughout the growth cycle of bacteria with a different concentration for each phase (15). It is a small basic protein (pI 9.5) composed of two subunits HU*α* and HU*β*. Each subunit has a molecular weight of 9.5 kDa. Crystallization studies have shown that the dimer is the fundamental unit and no higher order structures have been reported (16,17). In *E. coli*, HU exists as heterodimers (HU 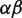) and homodimers (HU 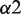 and HU 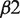). The heterodimer is the predominant form and has the highest binding affinity to double-stranded DNA (17). X-ray diffraction studies have shown that the heterodimers stack and wrap around DNA with an octameric repeat and induce superhelicity (18). Studies on the size of the HU binding site have reported values in the range of 9–42 bp/dimer, in a variety of conditions (19–21). Many of the proposed roles of HU have been attributed to the mechanical properties of the DNA–HU complex.

The bending rigidity of the DNA–HU complex has extensively been investigated before. Various single-molecule experiments have shown that the persistence length depends on the concentration of HU. DNA becomes more flexible at a relatively low concentration of HU, which has been attributed to HU-induced flexible bending of the duplex (21–25). At higher concentrations of HU, the DNA molecule is generally observed to become stiffer. This is attributed to the formation of a nucleoprotein filament with about one HU dimer per every nine base pairs (16,26). However, there is no quantitative agreement in the values of the persistence length at a particular HU concentration (23,24,27). Furthermore, another study has shown a decrease or at least no increase in persistence length above that of bare DNA at any HU concentration (28). Besides the concentration of HU, the mechanical properties of the DNA–HU complex also depend on the monovalent salt concentration in the supporting buffer. For instance, it has been reported that the above-mentioned variation in persistence length only occurs at a salt concentration <100 mM. At a higher concentration of salt, the persistence length is rather insensitive to the binding of HU on DNA (29).

Structural studies have shown that HU has a V-shaped body made of α helix with two protruding arms composed of β ribbons. The concave surface between the arms is exactly complementary to the right-handed double helix of DNA in the B-form. These arms interact with the duplex through intercalation between the base pairs (30). Intercalation results in a change in helical pitch of the duplex, which leads to supercoiling of closed circular DNA. The crystal structure studies of Anabaena HU bound to DNA has shown a dihedral bending angle that is consistent with negative supercoiling (31). Early gel electrophoresis and circular dichroism measurements have shown that HU induces supercoiling by a change in helical pitch of the duplex. The highest degree of supercoiling was observed when DNA was incubated with an equal mass ratio of HU (32,33). More recently, untwisting of the double helix by bound HU was confirmed in micromanipulation assays of single long DNA molecules (34). On the other hand, in an atomic force microscopy (AFM) study, it was shown that HU opens up, rather than interwind previously relaxed, closed circular DNA (35).

In most of the previous studies, the DNA molecules were incubated with HU for a relatively short time of <30 min. It is our contention that at least some of the contradictory results reported in the literature are related to rearrangement of bound HU on DNA over longer times. Accordingly, we have used AFM to monitor structural changes induced by HU on DNA molecules after an incubation time of various durations. AFM is particularly useful to study DNA–protein interaction because the complexes are directly visualized without an elaborate sample preparation procedure. Furthermore, the method is relatively free from artifacts related to aggregation, folding or bridging of the DNA molecules. Our study consists of two parts. In the first part, we measured the persistence length, height profile, contour length and bending angle distribution of the DNA–HU complexes after different incubation times. In the second part, we measured the change in topology of closed circular, relaxed plasmid following binding of HU. Eventually, we rationalize the various results in terms of unwinding of the duplex due to a longer-term structural reorganization of the nucleoprotein filament.

## MATERIALS AND METHODS

### HU protein purification

DNA plasmid pET Duet-1, which has been designed for co-expressing two genes, HU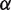 and HU*β* (HU*α* tagged with N-terminal his), was transformed into *E. coli BL21*. The cells were grown at 37°C in Luria broth medium containing ampicillin, and the overproduction of HU was induced by adding 4 mM IPTG at 20°C. The cells were lysed with a high pressure homogenizer. The lysate was cleared by centrifugation at 35 000 rpm for 30 min at 4°C. The lysate was then diluted with a buffer containing 250 mM NaCl, 10 mM Tris and 10% glycerol and loaded into a HisTrap HP column. The column was eluted with immidazole. The protein was further purified by using a Superdex 75 gel filtration column and dispersed in a buffer comprising 500 mM KCl and 10 mM Tris. The concentration was determined by ultraviolet absorbance at 230 nm with 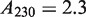 per 1 g of HU/l (36).

### Sample preparation

DNA fragments of 1000 bp and pUC19 (2686 bp) were purchased from Thermoscientific (Vilnius, Lithuania). DNA topoisomerase I, Vaccinia was purchased from Epicentre (Madison, Wisconsin). Plasmid was treated with topoisomerase I to obtain closed circular DNA. A total of 0.26 mg of DNA/l was incubated with different concentrations of HU in a buffer containing 20 mM HEPES, pH 7.5, 40 mM NaCl and 10 mM MgCl_2_ at 23°C. In all, 10 μl of the sample was then deposited on freshly cleaved SPI Grade V-4 mica without any further dilution. After 15 min, the mica was gently washed with deionized water, and blown dry with a stream of nitrogen gas (37,38). First, prior to imaging, DNA was incubated with different concentrations of HU for 2 h. Second, DNA was incubated with HU for different incubation times.

### Atomic force microscopy

The imaging was done at room temperature in air with a Nanowizard II atomic force microscope (JPK Instruments, Berlin, Germany). Images were acquired in the tapping mode with Nanosensor silicon (Si) cantilevers (spring constant of 10–130 N/m) and operated below their resonance frequency (typically 200–500 kHz).

**Figure 1.**
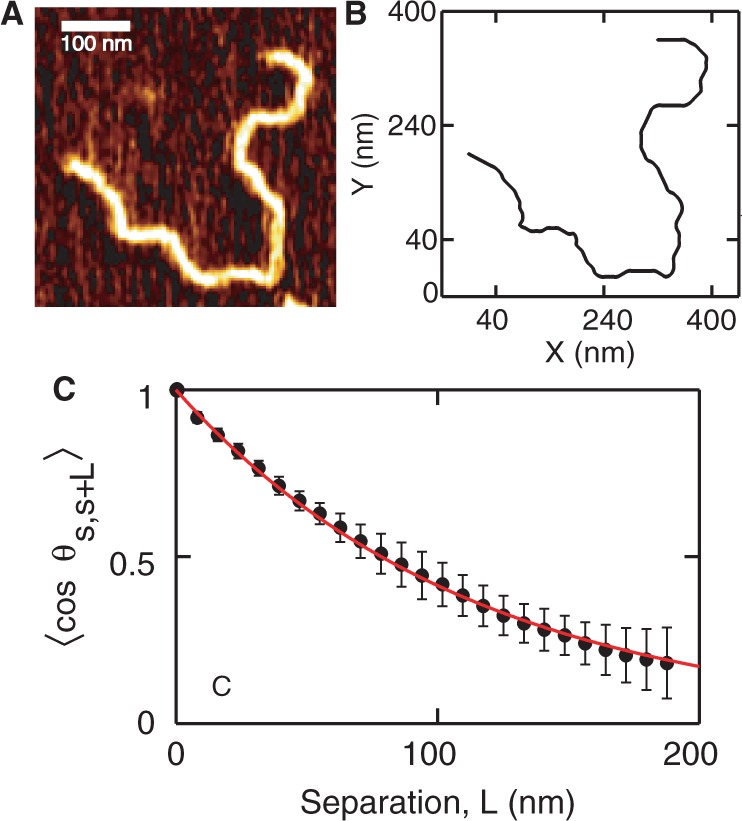
(**A**) A representative molecule of pUC19 equilibrated in 2D conformation on a freshly cleaved mica with 10 mM Mg^2+^. (**B**) The centerline of the DNA molecule is obtained by tracking the brightest point along the DNA contour. Each point on the centerline is separated by 8 nm. (**C**) Plot of 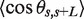 as a function of the separation *L*. The closed circles are the experimental data obtained by averaging at least 30 molecules. The experimental data are fitted using equation 1, shown by the red line. The fit gives a persistence length of 56 nm.

We used Mg^2+^ to bind the DNA molecules to the mica surface. This method allows the DNA molecules to equilibrate on the surface by diffusion. The interaction between DNA and mica mediated by Mg^2+^ is weak, so that the chain statistics is not affected (38,39). It has been shown previously that DNA deposited in this way equilibrates in a 2D conformation. To test our procedures, we imaged linearized pUC19 (2686 bp) deposited on mica in the presence of MgCl_2_. Figure 1A shows a representative molecule. We traced the contour with a step size of half the cross-sectional diameter (38). Thus, the data obtained are interpolated to get the centerline of the molecule as shown in Figure 1B. We then derived the tangent–tangent correlation, 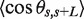, where *θ* is the angle between tangent vectors at points *s* and *s* + *L* on the contour. For DNA molecules equilibrated in 2D conformation, this correlation follows
(1)
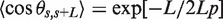

Hence, the inverse of the exponential decay constant gives the persistence length *L_p_*. Figure 1C shows the plot of the orientation correlation function averaged for ∼30 molecules. This curve is fitted using Equation (1). We obtained a persistence length 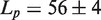 nm, which is in good agreement with values reported earlier (38–40).

## RESULTS AND DISCUSSION

### Change in persistence length with HU concentration

**Figure 2.**
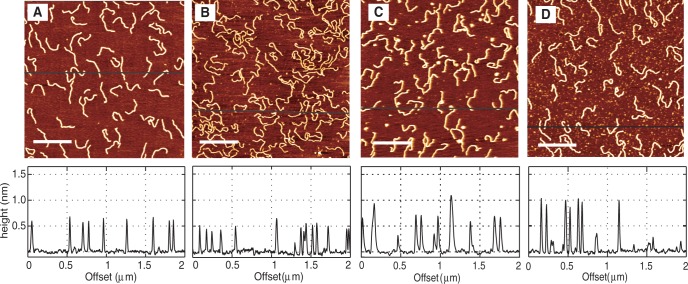
AFM images of 1000 bp DNA with no HU (**A**), 25 nM HU (**B**), 50 nM HU (**C**) and 900 nM HU (**D**) after an incubation time of 2 h. The dimer to base pair ratios in these cases are 1 dimer: 16 bp (B), 1 dimer: 8 bp (C) 1 dimer: 0.4 bp (D). The corresponding height profiles are shown below the respective images. The scale bars in the images are equal to 500 nm.

To study the persistence length and height of DNA, we have used short linear fragments of 1000 bp. DNA of this size allows visualization of structural changes with minimal effects related to spurious aggregation and folding. We first studied the persistence length as a function of HU concentration. Figure 2A shows 1000-bp DNA fragments in the absence of HU. A concentration of 0.26 mg of DNA/l and a sample deposition time of 15 min were used for all acquired images. In these conditions, we obtain a sufficient number of individual DNA molecules in a single frame for good statistics on the persistence length measurements. Because the molecules are equilibrated in 2D conformation, few DNA molecules, if any, are overlapping. Figure 2B–D shows the DNA molecules incubated with 25, 50 and 900 nM HU, respectively, for 2 h. The HU dimer-to-DNA base pair ratio at these HU concentrations is 1:16, 1:8 and 1:0.4, respectively. The height profiles are shown below the respective images. The height of bare DNA, as measured from the AFM images, is 0.5–0.7 nm. The height of the DNA–HU complex increased to ∼1 nm at 900 nM HU (Figure 2D).

**Figure 3.**
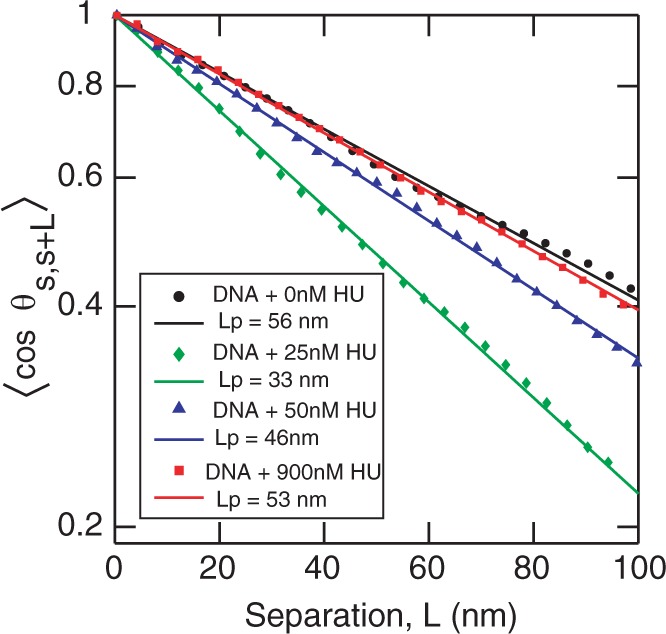
The correlation function 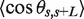 versus separation *L* of DNA without HU (black circles) and incubated with 25 nM HU (green diamonds), 50 nM HU (blue triangles) and 900 nM HU (red squares). The symbols are the experimental data obtained by averaging 30 molecules. The lines show the fitting to the experimental data using Equation (1).

Previous studies have shown that protein end-labeled DNA equilibrates on the surface of mica (39). We assume that the protein-coated DNA also equilibrates in a 2D conformation for the following two reasons. (i) We do not find any reduction in the length of DNA molecules as would be observed in the case of kinetically trapped molecules. (ii) There are few, if any, overlapping molecules. From the images, we derived the tangent autocorrelation function, 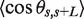 versus the separation *L* (Figure 3). The persistence length of DNA molecules without HU is obtained to be 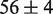 nm. The persistence length decreases to 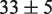 nm when incubated with 25 nM HU. However, with 50 nM and 900 nM HU, the persistence length increases again and becomes 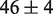 nm and 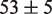 nm, respectively. The persistence length does not decrease further at lower HU concentrations. Notice that our samples have been incubated with HU for 2 h.

Our results show that there is a decrease in persistence length at a low HU concentration of 25 nM. There is almost no change in persistence length at higher HU concentrations, as compared with bare DNA. A possible reason for the insensitivity of the persistence length to higher HU concentrations is that HU does not bind to DNA. However, the height profile of the DNA molecules shows the contrary. The height of bare DNA, as measured from the AFM images, is 0.5–0.7 nm. The height of the DNA–HU complex increased to ∼1 nm at 900 nM HU, which indicates that the DNA molecule is coated by HU (panel D of Figure 2). Our observation is at odds with previous single molecule experiments, which showed that high concentration of HU results in an increase in persistence length (24,29). Deviations between our and previous observations can be related to the following issues: (i) we observed that the binding of HU to DNA depends on the absolute as well as the relative concentrations of HU and DNA. In our experiments, we fixed the concentration of DNA at 0.26 mg/l and the HU concentrations were varied. (ii) The interaction of protein with DNA and concomitant effects on the bending rigidity likely depends on the presence of MgCl_2_, which is necessary for adsorption on mica. (iii) The persistence length depends on the incubation time. Our samples were incubated for 2 h, whereas in previous works the molecules are usually imaged after a shorter time of ∼15–30 min.

### Time-dependent change in persistence length

**Figure 4.**
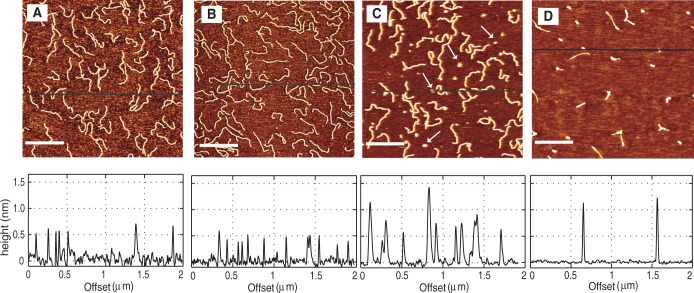
DNA molecules (1000 bp) incubated with 50 nM HU and imaged after (**A**) 15 min, (**B**) 1 h, (**C**) 2 h and (**D**) 21 h. The dimer to base pair ratio is 1:8. The corresponding height profiles are shown below the respective images. The scale bars denote 500 nm. Compacted molecules in panel C are indicated.

**Figure 5.**
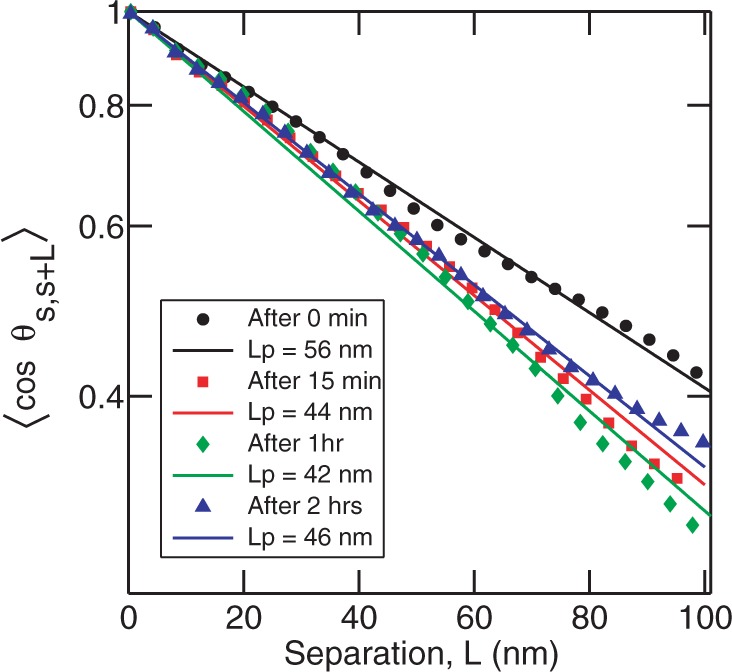
The correlation function 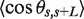 versus separation *L* of DNA incubated with 50 nM HU for incubation times of 15 min (red squares), 1 h (green diamonds) and 2 h (blue triangles). Black circles show the correlation curve for bare DNA. The symbols are the experimental data obtained by averaging 30 molecules. The lines show the fit to the experimental data using Equation (1).

To investigate a possible time dependence of the persistence length, we imaged the DNA–HU complex at higher HU concentrations (>1 dimer: 9 bp) following an incubation time of various duration. For this purpose, 0.26 mg of DNA/l was incubated with different HU concentrations between 50 and 900 nM. A droplet of sample was deposited on mica after different incubation times and, subsequently, imaged with AFM. The images and the corresponding height profiles are shown in Figure 4. Panels A, B, C and D show the DNA molecules incubated with HU and imaged after 15 min, 1 h, 2 h and 21 h, respectively. After 15 min of incubation (panel A), only a few HU dimers are bound to DNA. This can be concluded from the large amount of HU in the background of the image and from the height profile. There is no change in height when compared with bare DNA. After 2 h and as seen in panel C, almost all HU is bound to DNA. Here, two populations of DNA are observed, one which is compacted and another one which is not compacted. The non-compacted molecules exhibit a slight decrease in flexibility compared with bare DNA, and their height is ∼0.8 nm. The height of the compacted molecules is ∼1.5 nm. We observed that after 21 h, the molecules are all compacted (panel D). The persistence length of the compacted structures could not be determined. Figure 5 shows the graph of the orientation correlation function 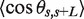 versus separation *L* obtained after different incubation times. The persistence length of the DNA–HU complex after 15 min, 1 h and 2 h of incubation is 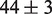, 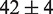 and 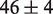 nm, respectively. There is a slight decrease in persistence length at 50 nM HU with respect to the value pertaining to bare DNA.

**Figure 6.**
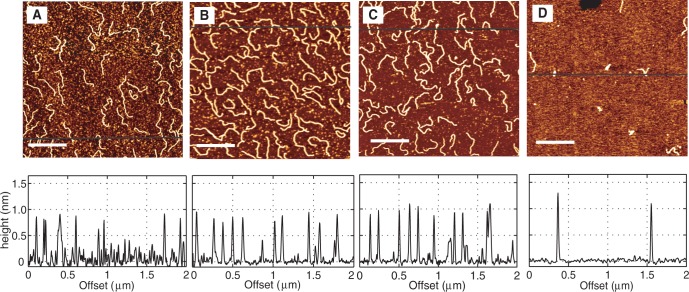
DNA molecules (1000 bp) incubated with 900 nM HU and imaged after (**A**) 15 min, (**B**) 1 h, (**C**) 2 h and (**D**) 24 h. The dimer to base pair ratio is 1:0.4. The corresponding height profiles are also shown. The scale bars denote 500 nm.

**Figure 7.**
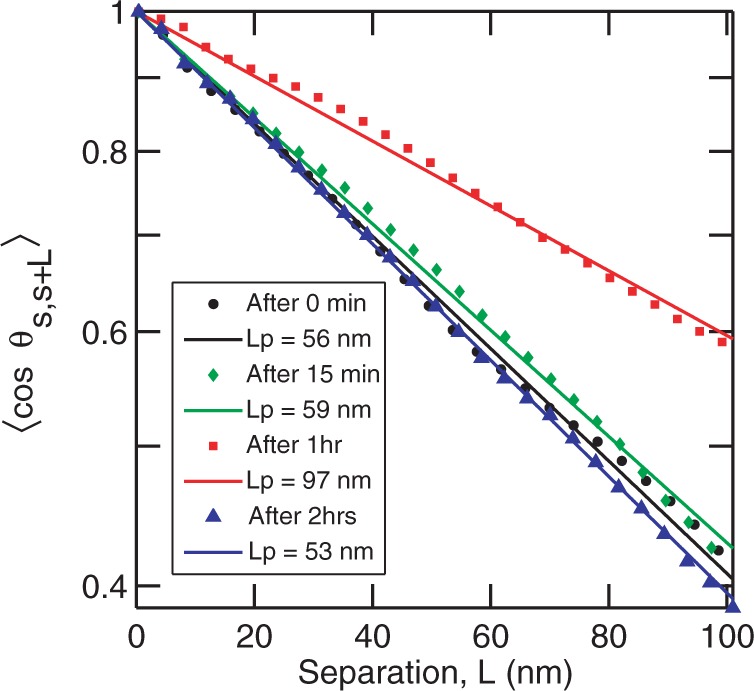
The correlation function 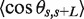 versus separation *L* for DNA molecules incubated with 900 nM HU for incubation times of 15 min (green diamonds), 1 h (red square) and 2 h (blue triangle). The dimer to base pair ratio is 1:0.4. The symbols are the experimental data obtained by averaging 30 molecules. The lines show the fit to the experimental data using Equation (1). Black circles show the correlation curve for bare DNA.

**Figure 8.**
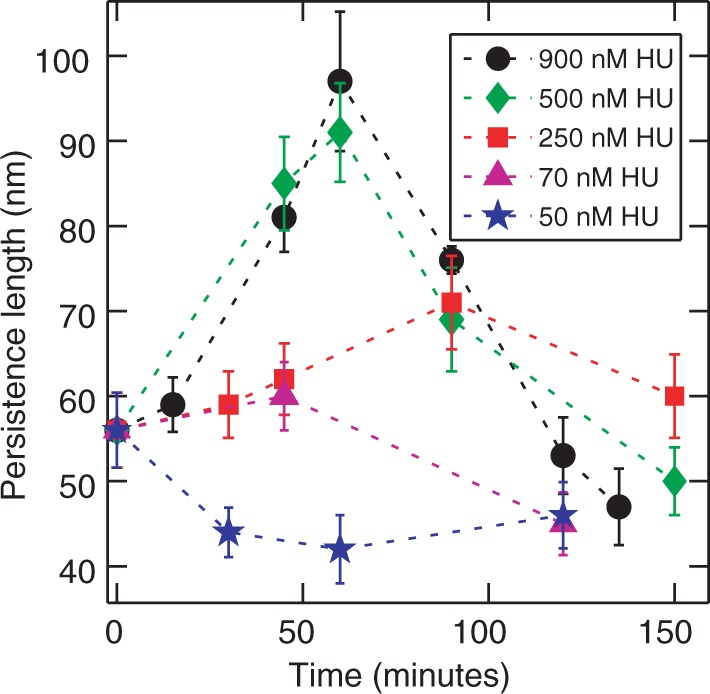
The persistence length as a function of incubation time for DNA incubated with different concentrations of HU. The concentrations of HU are 900 nM (black circles), 500 nM (green diamonds), 250 nM (red squares), 70 nM (pink triangles) and 50 nM (blue star). Each data point is obtained by averaging the values from three independent set of experiments with 30 molecules averaged in each case.

However, the time-dependent behavior of the persistence length is different at higher HU concentrations. For example, DNA was incubated with 900 nM HU and imaged after different incubation times. The dimer to base pair ratio at this concentration is 1:0.4. The images are shown in Figure 6. Panels A, B, C and D show the DNA molecules after incubation times of 15 min, 1 h, 2 h and 24 h, respectively. After 15 min of incubation, DNA molecules are not fully coated by HU. This is clear from the large amount of protein molecules in the background. The persistence length of these DNA molecules is determined to be 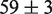 nm, as shown in Figure 7. Panel B shows the DNA molecules after 1 h incubation. There are fewer HU molecules in the background compared with that at 15 min, which indicates that more HU is bound to DNA. These molecules are stiffer with a persistence length of 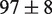 nm. After 2 h (panel C), almost all the protein molecules are bound. The corresponding height profiles have a uniform height of ∼1 nm. The persistence length reduces to 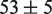 nm, which is the same as that of bare DNA. After 24 h, most of the DNA molecules are compacted as shown in panel D. Fewer molecules could be observed on the mica surface, as the binding of compacted DNA to mica is weak because of the decreased surface area and are easily washed off the surface during the sample preparation procedures. Duplicate experiments exhibit the same behavior, which is an initial stiffening followed by a reduction in persistence length to that of bare DNA. The time dependence of the persistence length with various HU concentrations is summarized in Figure 8. The dimer to base pair ratio at 50 nM HU is 1:8. This ratio can be considered as the onset of the high concentration regime. It has been reported that HU binds non-specifically to a 9 bp binding site at high concentrations of HU (20,22,31). In all aforementioned cases, 50 nM HU (1 dimer: 8 bp), a temporary stiffening is observed, followed by a reduction in persistence length to that of bare DNA.

For all cases, we observed compaction of the DNA molecules after 24 h. Because of the resolution limit of the AFM instrument, the internal structural arrangement of the compacted molecules cannot be determined. The compacted structures have a length of 45 ± 10 nm, a width of 17 ± 2 nm and a height of 1.5 ± 0.2 nm. These dimensions suggest that the molecules are condensed into a toroid, followed by an internal side-by-side collapse into a rod-like structure. With a total contour length of ∼340 nm, there are ∼5–6 loops of a single DNA molecule inside each compacted structure.

**Figure 9.**
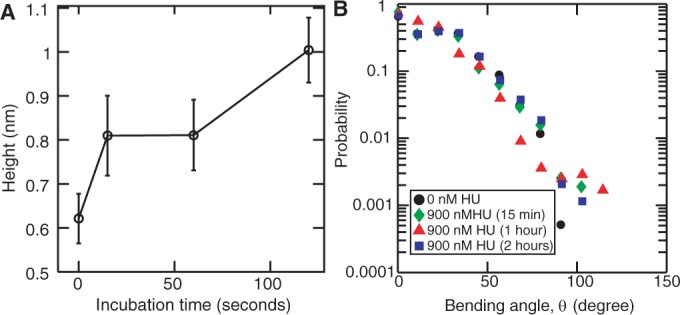
(**A**) Heights of the nucleoprotein filament versus the time of incubation with 900 nM HU. The value for zero incubation time pertains to bare DNA. (B) Bending angle distribution for bare DNA (black circles) and following incubation with 900 nM HU for 15 min (green diamonds), 1 h (red triangles) and 2 h (blue squares) pertaining to a separation along the contour of 10 nm.

There are three possible reasons for the observed decrease in persistence length with increased incubation time. (i) Progressive dissociation of protein could lead to a decrease in persistence length. However, the incubation time dependence of the measured height of the nucleoprotein complex is not consistent with such a phenomenon. Figure 9A shows the height for DNA incubated with 900 nM HU for 15 min, 1 h and 2 h. The heights were determined from the profiles taken in the transverse direction of the filament and averaged over 50 randomly chosen positions. After 2 h, the height of the filament is about two times the value pertaining to bare DNA, which indicates that the protein is still bound on DNA. (ii) Formation of kinks could lead to a reduction in persistence length. The importance of kinking can be gauged from an analysis of the bending angle distribution for a separation along the contour of a few nanometers on the order of the size of the binding site. Figure 9B shows the bending angle distribution for bare DNA and DNA incubated with 900 nM HU for 15 min, 1 h and 2 h and for a relatively short separation along the contour of 10 nm. The distributions were averaged along the contour and obtained for a pool of 30 molecules. We do not observe a significant change in bending angle distribution, besides the moderate effects related to changes in persistence length. Accordingly, binding of HU on DNA does almost certainly not result in the formation of sharp kinks. (iii) A change in helical pitch by binding of HU on DNA could affect the bending rigidity, and therefore the persistence length of DNA. Early circular dichroism measurements have indicated a change in helical pitch of dsDNA following binding of HU (32). More recently, unwinding of the duplex by bound HU has been observed in micromanipulation assays of single DNA molecules (34). Unwinding of the duplex should result in an increase in length of the nucleoprotein filament. Accordingly, we have also measured the contour length following incubation with 900 nM HU for different times.

### Time-dependent change in contour length

**Figure 10.**
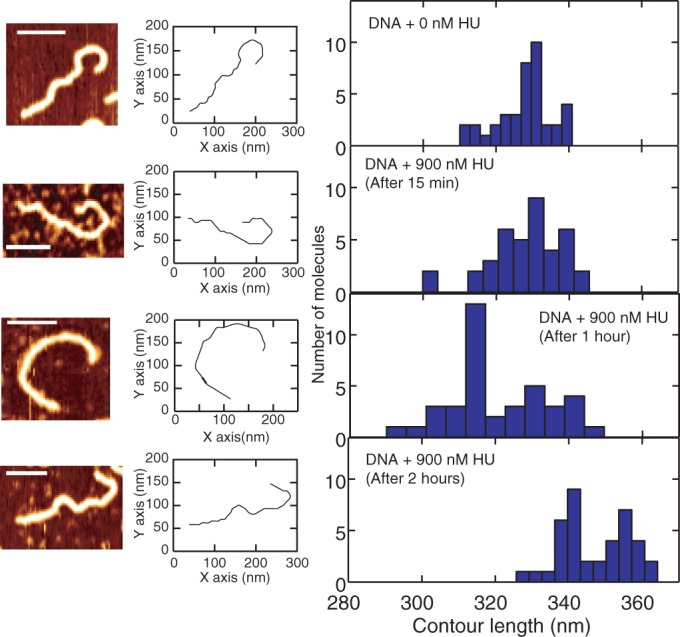
Representative AFM image, corresponding contour and contour length distribution for bare DNA and incubated with 900 nM HU for 15 min, 1 h and 2 h from top to bottom, respectively. The scale bars denote 100 nm. The distributions are obtained from a pool of ∼40 molecules each.

**Figure 11.**
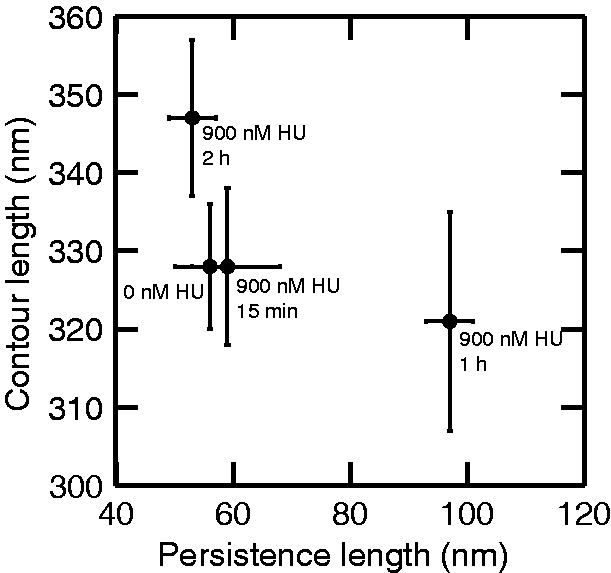
The contour length versus persistence length of 1 kb DNA molecules incubated with 900 nM HU for different incubation times.

Representative AFM images, contours and contour length distributions for bare DNA and DNA incubated with 900 nM HU for different times are shown in Figure 10. The contour length of bare DNA (1000 bp) was measured to be 328 ± 8 nm. When incubated with 900 nM HU for 15 min, 1 h and 2 h, the average contour lengths are 328 ± 10 nm, 321 ± 14 nm and 347 ± 10 nm, respectively. Accordingly, we observed a significant 6% increase in contour length once the molecules are incubated with 900 nM HU for 2 h. As shown in the correlation plot in Figure 11, the contour length is approximately inversely proportional to the persistence length.

### HU-induced supercoiling in closed circular DNA

Early circular dichroism, micromanipulation assays and our contour length measurements indicate unwinding of the duplex through intercalation of the protruding arms of HU between the stacked base pairs of the duplex (32,34). For closed circular DNA, such a change in helical twist of the duplex should result in a change in linking number deficit, and therefore superhelical density (41). In particular, relaxed closed circular DNA with zero linking number deficit should become supercoiled once the helical twist is modified.

**Figure 12.**
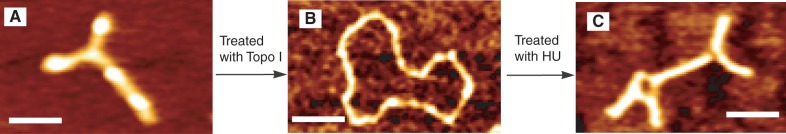
(**A**) Supercoiled pUC19. (**B**) Closed circular relaxed pUC19 after treatment of supercoiled pUC19 with Topo 1. (**C**) Supercoil after incubation of relaxed pUC19 with HU for 2 h. The scale bars denote 100 nm.

**Figure 13.**
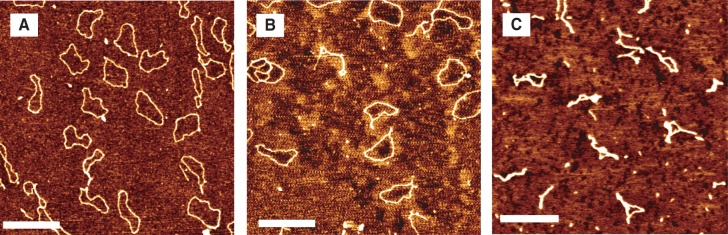
(**A**) Closed circular relaxed pUC19 after treatment with Topo 1. (**B**) Circular DNA incubated with HU in the ratio 1 dimer: 1 bp and imaged after 45 min and (**C**) 2 h. The scale bars denote 500 nm.

To visualize the supercoiling of DNA induced by binding of HU, we used relaxed closed circular DNA. For this purpose, pUC19 (2686 bp) was treated with topoisomerase I. This enzyme relaxes both left- and right-handed supercoils to obtain closed circular DNA as shown in panels A and B of Figure 12. These circular DNA molecules were subsequently incubated with HU in the dimer to base pair ratio 1:1. The images obtained right after treatment with topoisomerase I as well as following incubation with HU for 45 min and 2 h, respectively, are shown in Figure 13. We observed that there is no significant change in the circular topology of the DNA molecules after an incubation time of 45 min (panel B of Figure 13). Hence, for short-enough incubation time (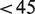 min), HU does not interwind and/or compact circular DNA. This observation agrees with the one made in earlier work (35). After a longer incubation time (2 h), the observed topologies are fundamentally different (panel C of Figure 13). Now, two main populations are observed: (i) circular molecules as in panels A and B of Figure 13, and (ii) branched interwound or side-by-side aggregated single molecular structures. The population of circular molecules with a percentage of ∼5% of the total is *de facto* open circles because one of the strands is nicked so that they cannot be supercoiled (the nicked duplex does not support twist). The other major population is most likely supercoiled and branched, rather than side-by-side aggregated. We measured an average length of the superhelical axis of these plectonemes and a contour length of the circular molecules being 360 ± 50 nm and 860 ± 40 nm, respectively. Hence, the ratio of the superhelical axis length to the DNA length is 0.42, which is in perfect agreement with the literature value of 0.41, irrespective of superhelical density (42). Thus, the interwinding of the relaxed closed circular DNA is due to the conversion of induced twist of the duplex into writhe.

### Mechanism

Our main observations can be summarized as follows. At a relatively low HU concentration that is corresponding to less than one dimer per nine base pairs, the persistence length decreases and takes a sub-bare DNA value of ∼45 nm for long incubation times. At higher concentrations of HU, the persistence length temporarily increases. The relative increase in bending rigidity depends on HU concentration, with the most pronounced effect for ∼1:1 dimer to base pair ratio. The apex in the persistence length occurs after an incubation time of ∼1 h. For longer incubation times, the persistence length decreases, and, after ∼2 h, the molecule regains the flexibility of bare DNA. Concurrently, we observed a 6% increase in contour length of linear DNA and interwinding of closed circular relaxed DNA after incubation with 900 nM HU for 2 h.

Binding of HU on DNA might result in a kink in the duplex or an increase in helical pitch because of intercalation of the protruding arms of HU (25,30). Neither our images nor the derived bending angle distributions show any kinks as compared with bare DNA. Single molecule studies have shown that under- or overwinding of the double helix leads to a reduction in persistence length (43). Hence, the observed reduction in persistence length for less than one HU dimer per nine base pairs is most probably because of a decrease in helical pitch of the duplex. The initial increase in persistence length following incubation with a high concentration of HU is related to the formation of a nucleoprotein filament. In this filament, HU is bound side-by-side with a spacing of nine base pairs or less along the contour. The increased bending rigidity might be caused by steric hindrance among the bound protein and/or electrostatic repulsive interaction. However, the subsequent decrease in bending rigidity and eventual regaining of the flexibility pertaining to bare DNA indicate a structural rearrangement of HU bound to DNA over a time span of ∼2 h. This rearrangement clearly involves a progressive unwinding of the duplex, resulting in an increase in bending flexibility of the filament. It should be noted that unwinding of the duplex results in an increase in linking number deficit. Relaxed closed circular DNA with zero linking number deficit interwinds and becomes positively supercoiled. However, *in vivo* DNA is negatively supercoiled with a negative linking number deficit. Binding of HU on negatively supercoiled DNA results in unwinding of the supercoil and a less compact molecular configuration. We surmise that this effect adds to the body of mechanisms, which controls the accommodation and expression of the genome inside the nucleoid of the bacterial cell.
